# Levonorgestrel-releasing intrauterine system for treatment of heavy menstrual bleeding in adolescents with Glanzmann’s Thrombasthenia: illustrated case series

**DOI:** 10.1186/s12905-018-0533-0

**Published:** 2018-02-27

**Authors:** Meiqiu Lu, Xin Yang

**Affiliations:** Gynecology and Obstetrics Department, Peking University People’s Hospital, Xi zhi men South Street 11, Xi cheng District, Beijing, PR100044 China

**Keywords:** Glanzmann’s thrombasthenia, Levonorgestrel-releasing intrauterine system, Heavy menstrual bleeding, Case report

## Abstract

**Background:**

Glanzmann’s Thrombasthenia (GT) is an inherited genetic disorder caused by defects in the platelet membrane glycoproteins IIb/IIIA, and is associated with heavy menstrual bleeding (HMB). HMB is a common complication in female patients, and many adolescent girls with this disease have issues with HMB beginning at menarche. The available treatment modalities including anti-fibrinolytics, nonsteroidal anti-inflammatory drugs (NSAIDs) and hormonal therapies though are effective, their associated side effects, limited efficacy and the poor compliance is a challenge in management of HMB. Levonorgestrel-releasing intrauterine system (LNG-IUS) has been a potential alternative to overcome this challenge. The use of the LNG-IUS for the management of HMB in adolescents with GT is explored in this case series.

**Case presentation:**

Two adolescents diagnosed with GT and received the LNG-IUS as treatment modality for management of HMB is discussed in this case series.

**Conclusions:**

For patients with poor compliance to oral hormonal therapies, the use of LNG-IUS is associated with a significant reduction of menstrual blood loss along with improved quality of life. These findings support the use of LNG-IUS to control adolescent GT-related HMB.

## Background

Glanzmann’s thrombasthenia (GT) is a rare inherited autosomal recessive bleeding disorder characterized by a lack of platelet aggregation and diminished clot retraction. It is caused by quantitative or qualitative defect in the platelet membrane glycoprotein IIb/IIIa (integrin αIIbβ3) [1, 2]. The disease is rare with an incidence of one in one million, but a relatively higher incidence is seen in populations where consanguineous marriages are common such as French Gypsies, Iraqi Jews, Jordanian Arabs, and South Indians [2, 3].. Quality of life in patients with GT is affected due to the spontaneous mucocutaneous bleeding and prolonged hemorrhage by trauma or surgery Purpura, epistaxis, gingival bleeding, and menorrhagia are the other common manifestations of this disease [1–5]. Heavy menstrual bleeding (HMB) clinically defined as blood loss of more than 80 ml per menstrual cycle is a debilitating social and health condition that has a negative impact on a woman’s physical, social, emotional, and material quality of life (QoL) [6, 7]. The available treatment modalities including anti-fibrinolytics, nonsteroidal anti- inflammatory drugs (NSAIDs) and hormonal therapies though proven effective, their associated side effects, limited efficacy and the poor compliance led to discontinuation, and this necessitate the requisite for alternative ‘fit and forget’ methods like levonorgestrel-releasing intrauterine system (LNG-IUS) [8, 9]. The safety and effectiveness of LNG-IUS in women with HMB are well demonstrated [10–12]. However, its use in GT-related HMB is not explored till date. Therefore, here we report first two cases of GT presenting with HMB where LNG-IUS was used for treatment of HMB.

## Case presentation

### Case 1

In 2010, a 14-year-old girl presented with a chief complaint of HMB for 1 year. The medical history reveals umbilical cord bleeding and frequent mucocutaneous bleeding events such as cutaneous purpura and ecchymosis after birth. As she experienced recurrent episodes of severe epistaxis, the girl was admitted to the local hospital several times for medical treatment at the age of five. The patient was diagnosed with GT and occasionally received blood transfusions. One year before her hospitalization at our ward, she experienced menarche with excessive menstrual bleeding that required blood transfusion. Since, her menstrual period always lasted more than 9 days and did not cease spontaneously, a platelet or erythrocyte transfusion (usually more than one per period) was needed to stop the bleeding. The family history revealed that she was the only child from her parents’ non-consanguineous marriage. Physical examination showed no abnormalities, except for generalized pallor. A gynecological examination by speculum showed moderate bleeding and laboratory investigation yielded the following results: leukocytes, 5.0 × 109/L; hemoglobin (Hb), 4.5 g/dL; and platelets, 155 × 109/L. The prothrombin time (PT) and partial thromboplastin time (PTT) were normal. An abdominal ultrasound showed uneven endometrial thickness of 7 mm. The patient first received oral contraceptive therapy for two courses each for 21 day cycles. After a course of treatment, the menstrual bleeding stopped, her anemia symptoms were relieved, and the Hb level rose to 8.8 g/dL. However, the patient repeatedly forgot to take the medicine during the course of therapy and this poor compliance caused irregular vaginal bleeding, ultimately leading to worse academic and social performance with a Hb level of 9.5 g/dL. After communicating with her parents, we offered her an alternative treatment option and placed a LNG-IUS in her uterus under general anesthesia. One month after the operation, her Hb level rose to 10.2 g/dL, menstrual blood volume had significantly decreased with a shorter period and symptomatic relief compared to the preoperative condition when measured using Pictorial Bleeding Assessment Chart Score (PBAC). Five months later, the patient was amenorrheic and no HMB episode has been reported since. An ultrasound examination showed an endometrial thickness of 4 mm, and the LNG-IUS was in a good location. At 2-year follow up after insertion of LNG-IUS, the Hb level was found to be 13.8 g/dL. In 2015, the girl came to our ward to have the exhausted LNG-IUS replaced, and her growth and development were good.

### Case 2

In 2014, a 14-year-old girl was admitted to our unit with a chief compliant of HMB during her second menstrual period. She had a history of severe gingival bleeding and epistaxis which led to hemorrhagic shock at the age of 8. At that time, she visited a local hospital and was diagnosed with GT. Her first period lasted only 3 days with less bleeding, while the second episode of menstrual bleeding started 8 weeks after menarche and required 10 tampons per day. The bleeding continued for 10 days, and the patient had symptoms of dizziness, weakness and mild abdominal pain. She was admitted to a local hospital where platelet (200 ml) and erythrocyte transfusion (400 ml) was infused to stop the bleeding. After basic treatment she was shifted to our hospital. She had no family history of bleeding disorders, and her parents have a non-consanguineous marriage. A physical examination revealed no abnormalities. Gynecological examination by speculum showed moderate bleeding. Laboratory examination revealed a normal platelet count; PT and PTT; while an abnormal platelet aggregation and altered hemoglobin level of 6.4 g/dL was observed. We recommended the LNG-IUS for this girl, but as her parents refused, oral contraception was prescribed to control her bleeding symptoms. The patient received oral contraceptive therapy (Marvelon) for five courses each for 21 day cycles. After the course of treatment, her Hb levels improved to 10.7 g/dL. However, during treatment, the child often forgot to take the pills. Given the poor patient compliance, the parents decided to allow the placement of a LNG-IUS, and the procedure was performed in 20 min under general anesthesia. However, the LNG-IUS fell out after the next menstrual cycle because of excessive menstruation. Therefore, she once again received oral contraceptive therapy. During the medication period, the girl had a severe headache, and we therefore, decided to place another LNG-IUS. Four months later, the patient was amenorrheic and her Hb levels rose to 12 g/dL. An ultrasound examination showed an endometrial thickness of 5 mm, compared to 8 mm prior to insertion of LNG-IUS. At the 2-year follow-up, she showed normal growth and development and had no complaints.

## Discussion

GT is a rare autosomal recessive bleeding disorder caused by either qualitative or quantitative abnormalities of αIIbβ3 integrin resulting from molecular genetic defects in *IFGA2B* or *ITGB3* genes [13]. It is characterized by faulty platelet aggregation and diminished clot retraction leading to excessive mucocutaneous bleeding and abnormally prolonged bleeding in response to injury or trauma. GT is rare disease with an incidence of one in one million and with slight predominance in women compared to men (58% vs 42%) [2]. Symptoms of the disease vary in severity from minimal bruising to severe and potentially fatal hemorrhages. The skin and mucosal bleeding such as petechiae, purpura and easy bruising are observable features at birth while, recurrent epistaxis, gingival bleeding and menorrhagia are the most common symptoms. In contrast, gastrointestinal bleeding, intracranial hemorrhage and hematuria are less common symptoms of the disease [3, 5]. This combination of clinical manifestations, the lack of platelet aggregation to physiologic agonists and diminished clot retraction along with a normal platelet count, size and morphology are he diagnostic criteria for GT [3, 5]. HMB is a common problem among women of reproductive age and is caused either by the immaturity of the hypothalamus-pituitary-ovarian axis or inherited bleeding disorders (IBD). HMB not only causes major health complications including iron deficiency anemia and the need for hospitalization and blood transfusion in severe cases, it can also adversely impact adolescent’s QOL, leading to loss of time from work and school, lifestyle and psychological disruption [7].

**Fig. 1:**
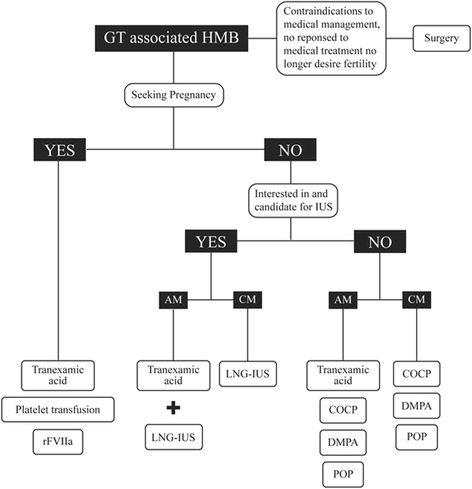
Proposed algorithm for medical treatment of GT-associated HMB in primary care. Abbreviations: AM, acute management; CM, continued management; COCP, combined oral contraceptive pill; DMPA, depot medroxyprogesterone acetate; LNG-IUS, levonorgestrel-releasing intrauterine system; POP, progesterone-only pill; rFVIIa, recombinant FVIIa

Anti-fibrinolytics, NSAIDs, hormonal therapies (combined oral contraceptive pills (COCPs), progesterone-only pills (POPs), depot medroxy progesterone acetate, danazol, gonadotrophin-releasing hormone analogs and LNG-IUS), platelet transfusion, recombinant FVIIa (rFVIIa) and surgery are the various modalities available for treatment of GT-related HMB [14–16] (Fig. 1). Although effective, anti-fibrinolytics proved to be unsuccessful in some cases and platelet transfusion was required if the bleeding was severe and persistent [5]. While, platelet transfusion controls bleeding in most patients, repeated transfusions will result in the generation of anti-platelet antibodies rendering platelet transfusion ineffectiveness. The use of rFVIIa is recommended in those who harbor anti-platelet antibodies [2]. Hormonal therapy with combined oral contraceptive pills (COCPs) and progesterone-only pills (POPs) should be used if the first-line therapy with anti-fibrinolytic is ineffective. COCPs and depot medroxyprogesterone acetate is also used for the continuous management of HMB in women with GT. LNG-IUS with or without the use of an anti- fibrinolytic agents has also been used to reduce bleeding. Surgical therapies such as hysterectomy or endometrial ablation are a therapeutic option for women who do not desire fertility [2].

The International Federation of Gynecology and Obstetrics (FIGO) gave a common terminology, abnormal uterine bleeding (AUB), for describing HMB and intermenstrual bleeding. They further developed the PALM-COEIN classification categorizing the etiologies for AUB as structural (PALM) and non- structural (COEIN) groups. The reason for HMB in adolescents were often non-structural in nature with anovulatory bleeding being the most common cause.

Although multiple maintenance treatments for HMB are available, LNG-IUS is a recommended option in adolescent females [17]. The LNG-IUS is a T-shaped intrauterine contraceptive device (IUCD) that releases progestin (20 μg daily) over 5 years at the local endometrium and has a significant effect on reducing menstrual blood loss by inducing endometrial gland atrophy, causing mesenchyme edema and inhibiting vascular proliferation. The safety and efficacy of LNG-IUS for treatment of high menstrual bleeding (HMB) is well demonstrated [10–12]. Andersson et al. studied the efficacy of LNG-IUS in 20 women with menorrhagia and found that the use of LNG-IUS resulted in a greater reduction in menstrual blood loss at follow up of 3 and 12 months [10]. Comparison of clinical effectiveness of LNG-IUS with common medical treatment (tranexamic acid, mefenamic acid, combined estrogen-progestogen or progesterone) revealed that LNG-IUS was more effective than medical treatments in reducing the effect of HMB on QOL [11]. In addition, a meta-analysis which compared the effects of the LNG-IUS with conventional medical treatment (mefenamic acid, tranexamic acid, norethindrone, medroxyprogesterone acetate injection, or combined oral contraceptive pills) also demonstrated that LNG-IUS was superior to medical treatments in reducing menstrual blood loss (weighted mean difference 136.00, 95% CI 74.43–197.57; *P* < .001). This study also demonstrated that the use of LNG-IUS is associated with higher rates of satisfaction (OR 5.19, 95% CI 2.73–9.86, *P* < .001), lower rate of discontinuation (OR 0.39, 95% CI 0.20–0.74), fewer treatment failures (OR 0.18, 95% CI 0.10–0.34) and improved QOL compared with conventional medical treatment [12]. In addition to these studies, LNG-IUS was also proven to be effective in the treatment of menorrhagia caused by IBD. Kingman et al. evaluated the use of the LNG-IUS in 16 women with IBDs and proved that LNG-IUS was effective and well-tolerated for menorrhagia treatment refractory to standard medical treatments [18]. Efficacy of LNG-IUS in 12 women with IBD at 3 and 6 months following insertion of LNG-IUS demonstrated a decrease in menstrual blood loss as well as improvement in the QoL [19]. Another study, reported LNG-IUS use had dramatically reduced menstrual blood loss and significantly improved the Hb concentration and QoL scores in 26 women with IBDs [19]. All these above studies demonstrate that effectiveness of LNG-IUS for treatment of HMB in women with IBDs. However, reports on the use of LNG-IUS for treatment of HMB associated with GT are scarce. Therefore, in this present study we report the use of LNG-IUS for treatment of HMB in two adolescent patients with GT who had failed other medical treatments and thus had an LNG-IUS inserted. The follow-up examination revealed a significant reduction in the menstrual blood loss and both patients were amenorrheic at 4–5 months after LNG-IUS placement. However, in the second case, the first LNG-IUS was spontaneously expelled during the next menstrual period; this might be due to heavy menstrual blood flow or uterine contractions or malposition of the device. Rimmer et al. [20] also reported an increased risk of expulsion or malposition of LNG-IUS in women with IBD than without a bleeding disorder and suggested that premedication or prolonged treatment with antifibrinolytics will reduce the risk of expulsion. Thus, the use of tranexamic acid during the insertion procedure and the subsequent one or two periods may reduce the risk of expulsion and if used along with LNG-IUS may have positive effect in future clinical applications.

## Conclusion

In summary, HMB is a common symptom in adolescent patients with GT. Active and proper treatment can promote physical and mental health and lead to better QoL. The LNG-IUS can remarkably shorten menstrual periods and reduce menstrual blood loss, with fewer systemic side effects. Moreover, it is associated with good compliance among teenage patients. Hence, it has considerable clinical potential for relieving mental and physical burden of GT-associated HMB in juvenile patients. However, large studies are warranted to understand the long-term effectiveness of the LNG-IUS for treating GT-related HMB and its advantages in comparison with COCPs.
